# Preoperative carbohydrate loading: evolution, trends, and future directions

**DOI:** 10.3389/fnut.2026.1750029

**Published:** 2026-03-11

**Authors:** Shuxia Liu, Qiaolan Liu, Yan Zhu, Ping Xiang, Xingyi Liu, Jianfei Cao, Miao Jia

**Affiliations:** 1 Department of Orthopaedics, Nanfang Hospital, Southern Medical University, Guangzhou, China; 2 Department of Comprehensive Ward, The First Affiliated Hospital of Jinan University, Guangzhou, China; 3 Department of Nursing, Dermatology Hospital, Southern Medical University, Guangzhou, China

**Keywords:** bibliometric analysis, Enhanced recovery after surgery, Insulinresistance, Management, Preoperative carbohydrate loading

## Abstract

**Background:**

Preoperative carbohydrate loading (PCL) counteracts the catabolic effects of fasting by alleviating insulin resistance and maintaining metabolic homeostasis. Despite its widespread use in clinical settings, there is no existing literature examining past achievements and breakthroughs in this area. The purpose of this study is to characterize its publication patterns, academic influence, research trends, and recent developments worldwide.

**Methods:**

The Web of Science Core Collection and PubMed were searched for documents published from the time the databases began to include relevant articles to March 27, 2025. Using VOSviewer, Citespace, and SciExplorer, a cross-sectional bibliometric analysis was conducted to extract and calculate evaluative indices. Publications were categorized by country, institution, author, journal, highly cited papers, and keywords. The variables were compared in terms of publication and academic influence, which also included citation count, journal impact factor, total link strength, collaboration metrics, and impact relative to the global community.

**Results:**

There were 269 publications, involving the contributions of 1,478 authors affiliated with 439 institutions across 38 countries/regions, and 141 clinical trials. China has the highest number of publications, albeit with limited collaboration, while England exhibits the highest centrality and a dense international cooperation network. The top ten journals in terms of publications are mostly high-quality journals. The author who has made the most outstanding and irreplaceable contribution is Olle Ljungqvist. Institutional cooperation is primarily geographically limited, with few links to transnational cooperation. We determine that “metabolic responses,” “recovery and clinical outcomes,” “preoperative management,” and “research techniques” are the most discussed research topics and identify four research frontiers and directions.

**Conclusion:**

Research on PCL has transitioned from early physiological validation to widespread clinical integration within ERAS protocols. The bibliometric patterns indicate that, despite substantial progress in understanding metabolic responses and perioperative management, key gaps remain in translating PCL evidence into personalized, harmonized, and technologically supported clinical nutrition practices. Clinicians and dietitians should prioritize tailoring PCL strategies to patient-specific factors, while integrating harmonized outcomes and technology to better assess individual responses. Besides, multidisciplinary collaboration between surgeons, anesthetists, and dietitians is essential to ensure consistent implementation.

## 1 Introduction

Major surgery triggers a systemic stress response characterized by pituitary and sympathetic nervous system activation, leading to profound metabolic disturbances, such as hyperglycemia, protein catabolism, and insulin resistance (1, 2). Preoperative fasting exacerbates surgery-induced insulin resistance, a pivotal metabolic alteration that impairs glucose uptake, reduces glycogen storage in both muscle and liver, and promotes muscle breakdown, ultimately leading to postoperative weakness (3, 4). More seriously, this insulin-resistant state is further amplified by the release of contra-insulin hormones, including glucocorticoids and catecholamines, which drive stress hyperglycemia—a phenomenon observed even in non-diabetic patients (5, 6). Clinically, insulin resistance has been demonstrated to correlate with increased morbidity, prolonged hospitalization, extended intensive care unit stays, and elevated mortality risks (7). These cascading metabolic derangements underscore the urgent need for preoperative interventions to mitigate the physiological toll of surgical stress.

Preoperative carbohydrate loading (PCL) is a crucial component of the enhanced recovery after surgery (ERAS) paradigm, which involves providing patients with carbohydrate-rich clear liquids for 2 h before elective surgery (8). By attenuating insulin resistance and preserving metabolic homeostasis, PCL counteracts the catabolic effects of prolonged fasting (9). This nutritional optimisation is synergised with intraoperative measures (e.g., blood glucose monitoring) and postoperative interventions (e.g., early nutrition), enhancing recovery trajectories and reducing healthcare costs, particularly in complex surgeries such as cardiac procedures (10–12). These benefits translate into an accelerated return of gastrointestinal function, reduced inflammatory reactions, and shorter hospital stays (13–15). Furthermore, PCL enhances patient comfort by alleviating preoperative anxiety and thirst (16, 17), while reducing postoperative nausea, vomiting, and pain severity (18, 19), without increasing the risk of aspiration when contraindications (e.g., gastroesophageal reflux) are appropriately excluded (14). Recent years have witnessed a growing academic interest in PCL studies, particularly regarding their potential to mitigate the adverse effects of overnight fasting (4, 7, 20). A preliminary analysis of the literature reveals that 184 peer-reviewed articles on PCL have been published in the last 10 years (2016–2025), compared to only 80 articles in the preceding decade (2006–2015), representing a 130% increase in research output. This surge in scholarly attention necessitates a systematic examination of the field's evolution and emerging patterns. Despite the proliferation of research in this domain, a notable gap persists in comprehensive bibliometric analyses of PCL. There is an urgent need for an extensive review and analysis of the existing literature to understand better the past achievements and breakthroughs in this area, as well as their implications in the perioperative period. It should be noted that this bibliometric analysis focuses explicitly on carbohydrates rather than mixed or carbohydrate-protein formulations in preoperative nutrition. Firstly, carbohydrate solutions alone have consistently been the primary interventions studied in perioperative nutrition research (21). The mechanistic basis for pure carbohydrate loading centers on its specific capacity to alleviate surgery-induced insulin resistance and preserve glycogen stores, without the gastric-emptying delays associated with formulations containing protein or fat. Secondly, from a safety perspective, clear carbohydrate solutions can be safely administered within 2 h pre-anesthesia according to established fasting guidelines (8). In contrast, mixed macronutrient beverages may necessitate longer fasting periods due to delayed gastric emptying. This practical advantage has established pure carbohydrate solutions as the standard intervention in ERAS and the primary focus of most clinical trials. Finally, the physiological endpoints most frequently studied in PCL research—including insulin sensitivity, glucose homeostasis, and metabolic stress responses—are best addressed through carbohydrate-specific interventions rather than mixed formulations, where the individual contributions of carbohydrates and other macronutrients become difficult to disentangle. Whilst carbohydrate-protein combinations may offer additional benefits, these represent distinct interventions with differing mechanisms and safety profiles, warranting separate meta-analyses. Thus, we applied mathematical and statistical techniques to evaluate and quantify the literature in the PCL field. Our study aims to address three principal research questions:

1. What constitutes the current research landscape regarding geographical distribution (countries/institutions), publication venues (journals), and influential contributors (authors/references) in PCL studies?;
2. What are the predominant research themes in this field?;
3. What emerging trends and potential future research directions can be identified within PCL research?.

## 2 Methods

### 2.1 Data acquisition and search strategy

The raw data analyzed in this study were retrieved and exported on March 27, 2025, from the Web of Science (WoS) Core Collection (WoSCC) and PubMed. WoS was prioritized as the principal data repository due to its comprehensive inclusion of more than 12,000 rigorously vetted journals and its well-documented reliability in bibliometric studies (22). To ensure comprehensive and accurate retrieval, a truncation-based search strategy using wildcards (e.g., ^*^) was applied in WoS. The search strategy was set as: (((((TS=(pre$operat^*^)) OR TS=(pre$surgical)) OR TS=(peri$operat^*^)) OR TS=(surg^*^)) OR TS=(operat^*^)) AND (((TS=(carbohydrate$)) OR TS=(CHO)) OR TS=(maltodextrin)) AND (((((((TS=(load^*^)) OR TS=(drink^*^)) OR TS=(beverage$)) OR TS=(treatment$)) OR TS=(administration$)) OR TS=(oral)) OR TS=(per os)). Search strategy for PubMed is shown in Supplementary Material. This two-database approach ensured comprehensive coverage of both basic research and clinical trial literature for further analysis. Given the inherent differences between WoS and PubMed in terms of indexing standards, clinical tagging systems, and metadata structures, directly merging data from both sources could compromise the comparability and scientific validity of the bibliometric results. Therefore, this study adopted a “separate database analysis plus integrated discussion” approach (23). WoSCC data were mainly used to track the evolution of basic and high-impact research, while PubMed data focused on analyzing clinical trial literature, serving as a supplement to evaluate translational potential and clinical implementation strategies. In terms of document types, the WoS search was restricted to “Articles” and “Reviews,” while the PubMed search was refined to include studies categorized as “Clinical Trials”. No philological constraints were applied during the search to minimize potential distortions from linguistic preferences. As shown in Table 1, the two manual screening criteria were content and quality. The content criteria guaranteed the publication's relevance to the study's topic, while the quality criteria ensured that the article adhered to basic academic paper formatting standards. The following records were excluded from the analysis: (1) publications including early access, retracted ones, proceeding papers, meeting abstracts, editorial materials, book chapters, letters, correction notices, and those with expressions of concern; (2) duplicate records; and (3) publications with incomplete or missing bibliographic information. Two reviewers independently conducted the inclusion and exclusion processes. In cases of disagreement, a third reviewer was consulted to resolve discrepancies and reach a consensus.

**Table 1:** Inclusion criteria for manual screening.

**Type**	**Inclusion criteria**
Content criteria	The included literature should focus on the preoperative carbohydrate loading, with other research on carbohydrate loading not being considered for inclusion in the study.
	Studies conducted only on animal models and no human experiments or work have been conducted
Quality criteria	The included literature consisted of at least two pages, and literature below this threshold was excluded.
	The literature must contain essential elements of an academic paper, such as abstracts, author information, keywords, and references. Any literature lacking any of these elements will be excluded.
	The literature undergoes a standardized double-blind peer review process.

### 2.2 Data cleansing, extraction, and standardization

The data cleansing process was conducted on the WoSCC and PubMed websites, respectively. The retrieved records were exported in plain text format, and preprocessing involved removing special characters and redundant whitespace. To prevent omissions in manual screening, the filtered literature was imported into Citespace to eliminate duplicate literature. Furthermore, singular, plural, and synonymous forms in the keyword field were merged. Data extraction, cleansing, and standardization were conducted independently by two researchers and cross-validated; any ambiguity was resolved through consensus by a third researcher. This methodological framework, formulated through extensive consultation with senior information retrieval specialists and unanimously ratified by all contributing authors, adheres to established best practices for macroscopic bibliographic evaluations.

### 2.3 Software for bibliometric analysis

The bibliometric analysis utilized CiteSpace (v6.3. R1), VOSviewer (v1.6.20), and SciExplorer to appraise publications addressing PCL systematically. CiteSpace is a Java-based bibliometrics application developed by Chaomei Chen, aiming to visually map highly cited and pivotal documents and analyse the emergence of research topics (24, 25). It constructs network visualizations (co-citation networks, collaborative authorship matrices, and keyword burst detection) to elucidate structural and chronological patterns, wherein nodal elements denote academic entities (authors, institutions, and nations) and edge thickness signifies collaboration intensity. Centrality metrics (indicated by violet-encircled nodes) identify seminal contributions. VOSviewer is another freely available program that facilitates co-occurrence mapping and collaborative network analysis, employing proportional node sizing to represent keyword prevalence and chromatic gradients to chronicle temporal progression (26, 27). It is particularly valuable for displaying large bibliometric maps in a clear and easy-to-interpret way. SciExplorer (https://smartdata.las.ac.cn/SciExplorer/?lang=CN) is an open online scientometrics data analysis and visualization system, developed by the National Science Library, Chinese Academy of Sciences, China. It is widely praised for its openness, security, professionalism and intelligence. Integrating scenarios and technology enables it to serve the scientific community better.

### 2.4 Visualization analysis

This analysis encompassed various aspects, including countries, institutions, authors, journal distribution, references, and keywords. VOSviewer v1.6.20 primarily facilitated the study of co-occurrence and clustering of authors and keywords. Different nodes represented different elements, and various colors in the co-occurrence maps indicated the differences in clustering. CiteSpace v6.3. R1 was used to analyse the co-occurrence and clustering of countries, institutions, and keywords, as well as the dual-map overlay of journals and bursts of keywords and references. SciExplorer provided the chord diagram of countries and institutions. The journal impact factors were retrieved from the Journal Citation Reports (JCR) of 2023 in WoSCC. Additionally, primary data set management and exponential growth projections were conducted using Microsoft Excel.

### 2.5 Parameter setting

The documents were imported into CiteSpace for co-occurrence and clustering analysis. The time slice for keyword nodes was 1 year, and the threshold value was Top N = 50, indicating that the top 50 most frequently occurring keywords from each year were filtered out to construct the co-occurrence network. Subsequently, individual networks were synthesized. The networks were pruned using the “Pathfinder network” and “Pruning sliced network” methods to eliminate redundant nodes and connections. The modularity score Q (ranging from 0 to 1) was used to assess the network's structure, with a value exceeding 0.3, indicating a well-structured network. The silhouette score S (ranging from −1 to 1) was utilized to evaluate the trustworthiness of clustering mapping. Values above 0.5 indicate reasonable clustering, while those exceeding 0.7 signify a highly trustworthy network (28). The dual-map overlay analysis in CiteSpace was conducted using default parameters. In VOSviewer, adjustments were made to the number of nodes in the graph, while the remaining parameters remained at their default values.

### 2.6 Ethics and reporting

**Figure 1:**
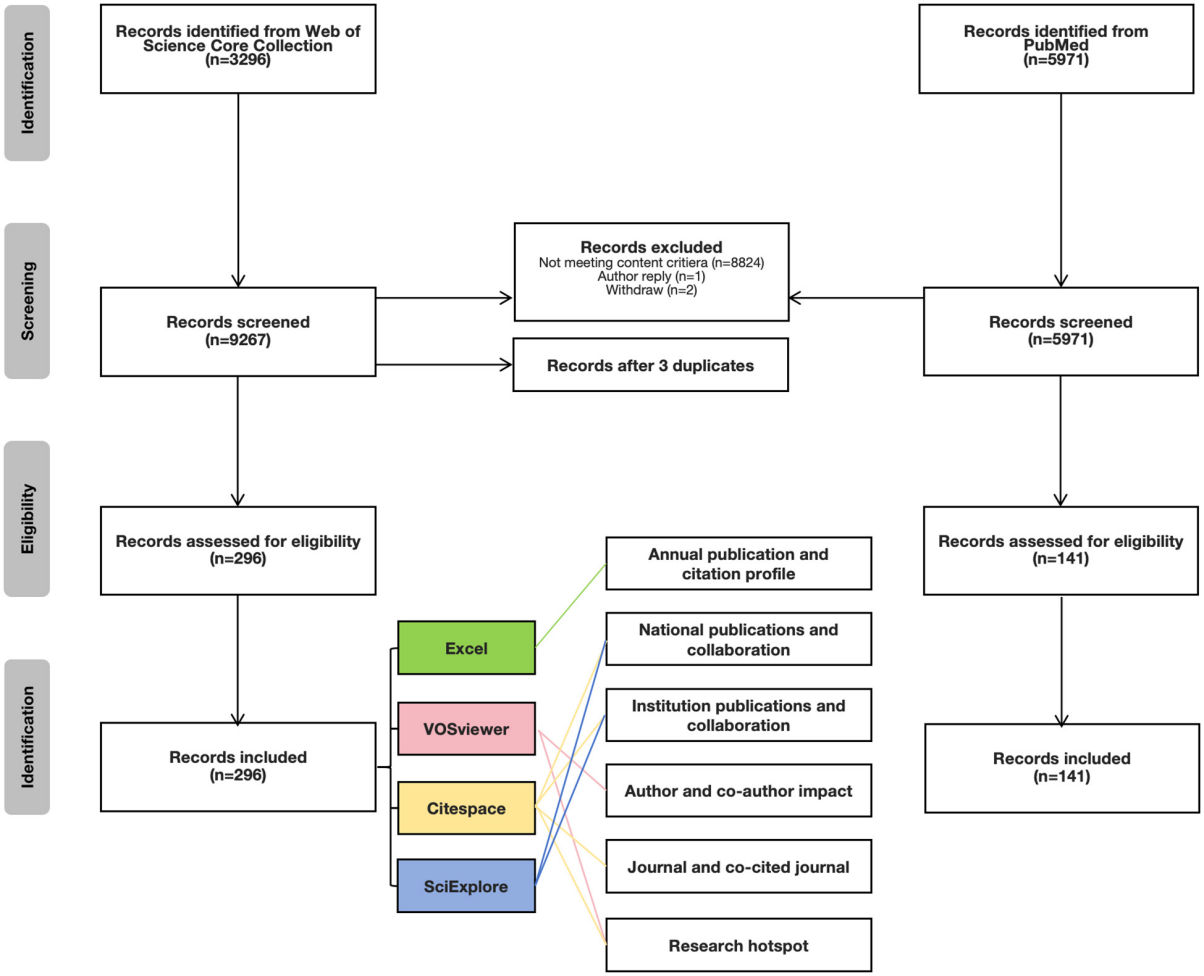
Flowchart of the study.

Since the data originated from the publicly accessible WoSCC and PubMed databases, obtaining ethical approval from an institutional review board was deemed unnecessary. This study adhered to the Preferred Reporting Items for Systematic Reviews and Meta-Analyses (PRISMA) 2020 guidelines, and a PRISMA flow diagram was provided to illustrate the search strategy and bibliometric analysis (Figure 1).

## 3 Results

### 3.1 Database search

According to the search strategy, 269 publications were retrieved from the WoSCC database. These publications involved the contributions of 1,478 authors affiliated with 439 institutions across 38 countries/regions. They were published in 120 journals and referenced 4,672 articles from 1,513 journals.

### 3.2 Annual publication volume, citation volume and trends

**Figure 2:**
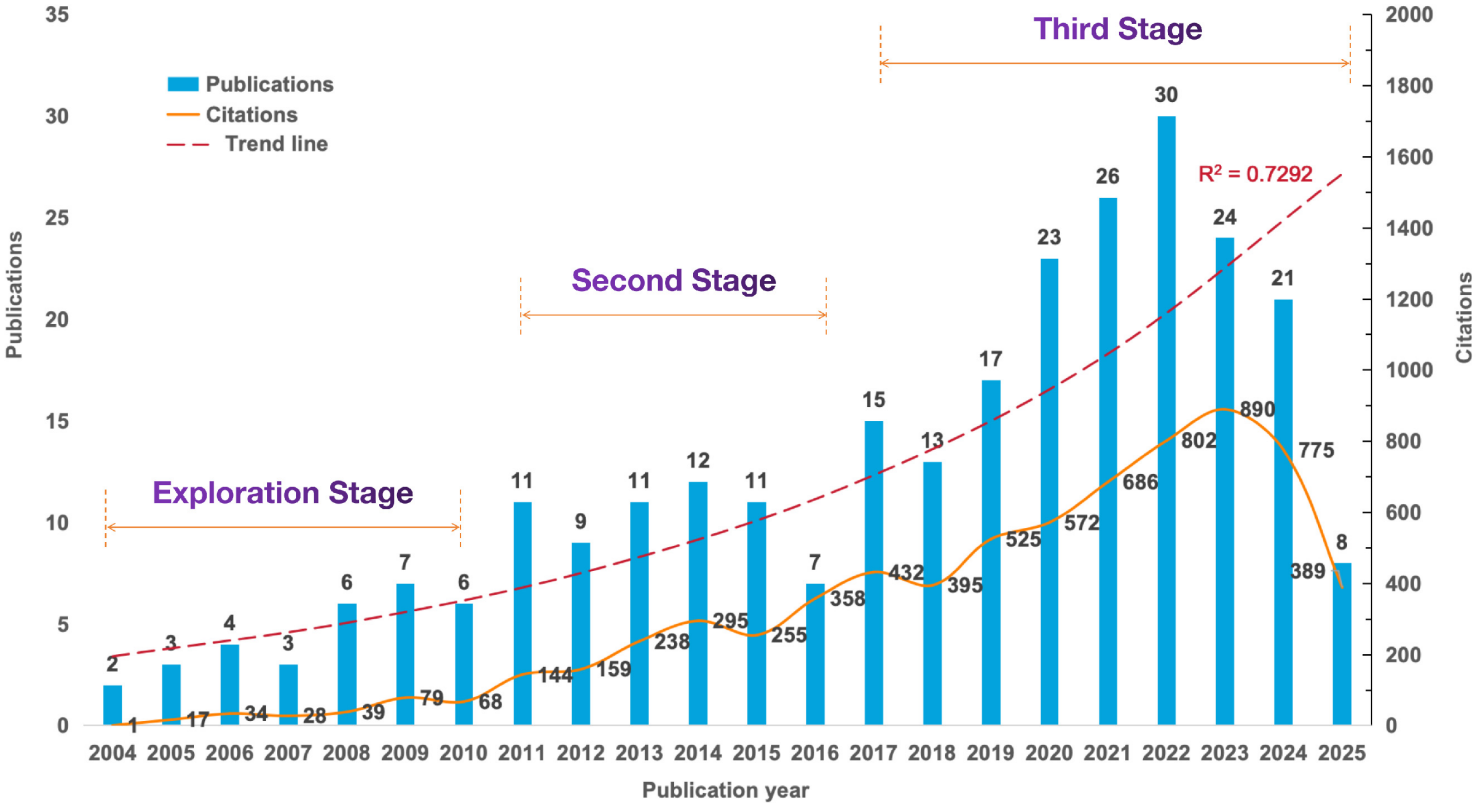
Trends in the annual publication and citation of articles on preoperative carbohydrate loading between 2004 and 2025.

The annual publication volume and citation trends were shown in Figure 2, with the earliest articles in PCL dating back to 2004. The maximum number of articles was 30 in 2022, whilst the maximum number of citations reached 890 in 2023. The number of publications and citations demonstrated a statistically significant relationship with the year, as evidenced by binomial fitting analysis (publications, *R*^2^ = 0.7292; citations, *R*^2^ = 0.7748). Except for a slight decline in 2016, the number of publications over the past 22 years has shown an overall upward trend, manifesting in three distinct periods. Between 2004 and 2010, research on PCL was in its embryonic stage, with an annual publication volume ranging from 2 to 7, averaging 4.4 publications per year. From 2011 to 2016, the yearly publication volume was relatively stable (between 9 and 12), with a sudden drop in 2016 (n = 7), but overall it was twice as high as the previous decade. The third period, from 2017 to 2025, accounted for 62.82% of total publications. The growth rate of publications has increased rapidly in this stage, and the annual number of publications began to stabilize at 20 or more after 2020. Citations have been fluctuating upwards and peaked in 2023 before declining.

### 3.3 National publications and collaboration

**Figure 3:**
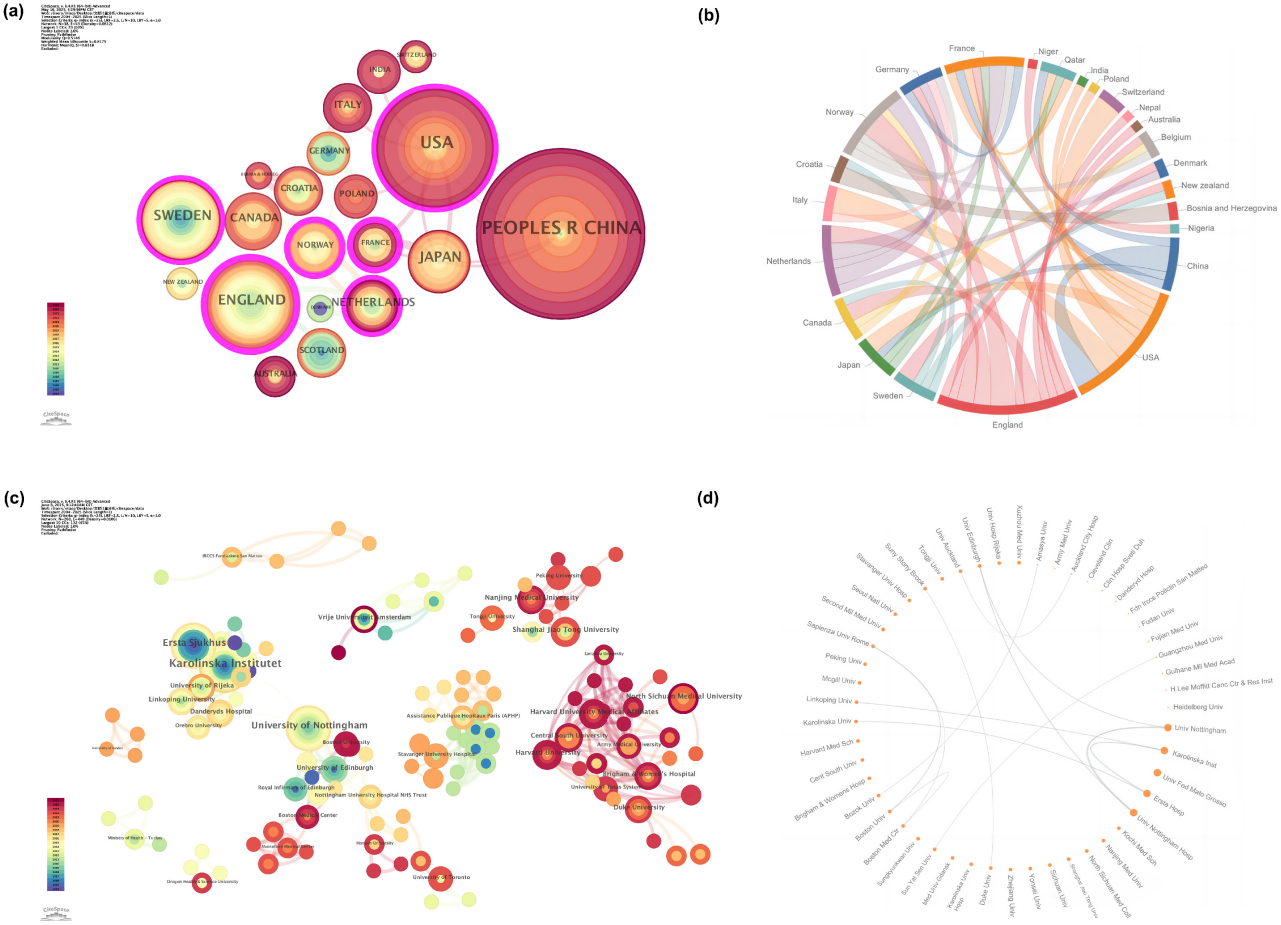
The collaboration of countries/regions and institutions in preoperative carbohydrate loading. **(a)** The collaboration network of countries/regions; **(b)** Chord diagram that visualizes countries/regions‘ collaboration; **(c)** The collaboration network of institutions; **(d)** Circos plot that visualizes institutions' collaboration.

The collaboration network of countries/regions was generated to identify their scientific research contributions and cooperation relationships in this field (Figures 3a, b). The top ten countries contributed the most, accounting for 82.90% of publications (Table 2). The People's Republic of China emerged as the leading contributor, with 62 papers (23.05%), more than twice the number of publications from the United States of America (USA) (35 papers, 13.01%). Following closely were England (23 papers, 8.55%) and Sweden (22 papers, 8.18%). These top four countries published 142 papers on the topic of interest, accounting for 52.79% of the whole publication pool. England (0.31), France (0.3), Norway (0.23), the USA(0.19), the Netherlands (0.15), and Sweden (0.12) were in descending order of centrality. Notably, France (0.3, *n* = 4) and Norway (0.23, *n* = 6) had the second-highest and third-highest centrality, despite having fewer publications. The collaboration network (Figures 3a, b) showed that the USA, England, and Sweden had large nodes and dense connectivity, indicating high national cooperation. Compared to Norway, the Netherlands, and France, which had smaller node countries with greater international cooperation, China had a large node but few connections, indicating a lack of collaboration and limited connections. The number of publications from Asian countries, while commendable, was relatively limited in terms of cooperation with others. When considering the top five countries in terms of citations, England (2,069 citations) outperformed other nations, followed by Sweden (1,429), the Netherlands (684), the USA (571), and China (527). Additionally, England had the highest number of citations per article (89.96), while China had the lowest (8.50).

**Table 2:** The top 10 countries' contribution and collaboration to PCL.

**Rank**	**Country**	**Documents**	**Centrality**	**Citations**	**Average citations**
1	China	62	0	527	8.50
2	USA	35	0.19	571	16.31
3	England	23	0.31	2,069	89.96
4	Sweden	22	0.12	1,429	64.95
5	Japan	17	0	202	11.88
6	South Korea	16	0	166	10.37
6	Turkey	16	0	344	21.50
8	Brazil	14	0	410	29.29
9	Canada	9	0	331	36.78
9	Netherlands	9	0.15	684	76.00

### 3.4 Institution publications and collaboration

According to Table 3, the top ten most productive institutions contributed 79 articles, accounting for 29.37% of the total articles. The University of Nottingham in England contributed the most (*n* = 10, 3.72%), followed by Karolinska Institutet in Sweden and the Federal University of Mato Grosso in Brazil, which published nine papers respectively. More than half of the top 10 institutions in terms of output were located in Asian countries, and up to 75% of the top 10 institutions were universities. The institution-level collaboration network was mapped to explore influential institutions and analyse their collaboration degree (Figure 2c). The network consisted of 288 nodes and 440 links, with a network density of 0.0106. The centrality of institution collaboration was all less than 0.1, with “Lanzhou University” (0.02) and “Shanghai Jiao Tong University” (0.02) being the top two. The University of Nottingham collaborated most frequently with other institutions (frequency: 10, links: 2), while Karolinska Institutet collaborated with the most significant number of institutions (frequency: 9, links: 5) (Figure 2d). Overall, the research institutions were scattered and lacked extensive and consistent cooperation. As for the average citations, Ersta Hospital stood alone and far exceeded those of other institutions. Regrettably, although institutions in Asian countries published a considerable number of articles, the average number of citations for these articles was less than 15 times, except for Shanghai Jiao Tong University.

**Table 3:** The top 10 institutes in the field of PCL.

**Rank**	**Organization**	**Country**	**Documents**	**Citations**	**Average citations**
1	University of Nottingham	England	10	317	31.70
2	Karolinska institutet	Sweden	9	591	65.67
2	The Federal University of Mato Grosso	Brazil	9	356	39.56
4	Nottingham university hospitals	England	8	466	58.25
4	Ersta hospital	Sweden	8	801	100.12
6	Kochi medical school hospitals	Japan	5	58	11.60
6	Nanjing medical university	China	5	20	4.00
6	North sichuan medical college	China	5	20	4.00
6	Shanghai jiao tong university	China	5	165	33.00
6	Sichuan university	China	5	32	6.40
6	Yonsei university	Korea	5	69	13.80
6	Zhejiang university	China	5	45	9.00

### 3.5 Author and co-author impact analysis

**Figure 4:**
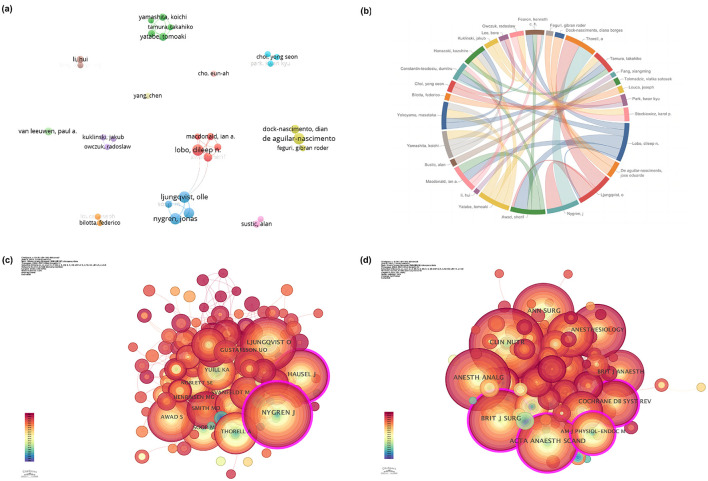
The collaboration of authors, co-authors, and co-journals in preoperative carbohydrate loading. **(a)** The collaboration network of authors; **(b)** Chord diagram that visualizes authors' collaboration; **(c)** The collaboration network of co-authors; **(d)** The collaboration network of co-journals.

Based on the analysis of the literature retrieved, 1,478 authors and 3,785 co-cited authors were involved in the study of PCL. Table 4 presents the top ten productive authors in this field. It could be intuitively seen that Olle Ljungqvist, Jonas Nygren, Dileep N. Lobo, and José Eduardo de Aguilar-Nascimento tied for the most productive authors, all of whom published 10 articles. According to Lodka's Law, the number of core authors who published three or more papers is estimated to be 33. Figures 4a, b showed their collaboration on this research topic. Although the collaboration network among authors presented some obvious team cooperation relationships, the overall cooperation was insufficient (centrality was all less than 0.02). Dileep N Lobo collaborated with other authors the most times and with the most authors (Frequency: 17, Links: 5), followed by Olle Ljungqvist (Frequency: 16, Links: 4). In terms of average citations, only M Soop and Jonas Nygren published articles with an average citation greater than 100. Among the top ten co-cited authors (Supplementary Table S1), Jonas Nygren was the only author in the top ten with more than 300 co-cited records (308), followed by Olle Ljungqvist (245) and J Hausel (178). Jonas Nygren (0.16) and J Hausel (0.14) were the only two authors with a centrality greater than 0.1, highlighting their outstanding contributions in this field. Surprisingly, 60% of the most productive co-authors and 60% of the top five authors were from Sweden.

**Table 4:** The top 10 authors in the field of PCL.

**Rank**	**Author**	**Documents**	**Citations**	**Average citations**	**Organization**	**Country**
1	Olle Ljungqvist	10	933	93.3	Örebro University/Karolinska Institutet	Sweden
1	Jonas Nygren	10	1,001	100.1	Karolinska Institutet	Sweden
1	Dileep N Lobo	10	516	51.6	Nottingham University Hospitals	United Kingdom
1	José Eduardo de Aguilar-Nascimento	10	374	37.4	Federal University Of Mato Grosso	Brazil
5	Anders Thorell	9	719	79.889	Karolinska Institutet	Sweden
6	Sherif S Awad	6	359	59.833	Minia University	Egypt
6	Diana Borges Dock-Nascimento	6	255	42.5	Santa Rosa Hospital	Brazil
8	Tomoaki Yatabe	5	58	11.6	Fujita Health University School	Japan
8	Paul van Leeuwen	5	101	20.2	Vu University Medical Center	Netherlands
10	Ian A Macdonald	4	139	34.75	Nottingham University Hospitals	United Kingdom
10	Mattias Soop	4	430	107.5	Karolinska Institute	United Kingdom
10	Koichi Ando	4	55	13.75	Showa University School	Japan
10	Masataka Yokoyama	4	31	7.75	Kochi Medical School	Japan
10	Kenneth C H Fearon	4	155	38.75	The University Of Edinburgh	United Kingdom
10	Alan Sustic	4	43	10.75	University Of Rijeka	Croatia

### 3.6 Distribution of journals and co-cited journals

From 2004 to 2025, 120 journals published articles related to PCL. When attempting to establish the Bradford zones, we observed that there was no distinct level of publication that served as a criterion for dividing the set of journals into three. In this case, we opted for an elitist criterion, understanding that a core of 10 journals contained nearly 30% of the articles (29). Table 5 details the top 10 journals in this research field. Web of Science reported the 2023 JCR and Impact Factor (IF). Three journals with an IF of more than four were in the Q1 JCR division, five were in the Q2 JCR division, and the remaining two were in the Q3 and Q4 JCR divisions, respectively. *Clinical Nutrition* published the highest number of articles (16 papers, IF: 6.6). Although the *British Journal of Surgery* just published six papers, it had the highest IF (8.7), total citation (752), and average citations (125.33). These two journals were the only ones with an average of more than 50 citations. Additionally, Wiley was the top-ranked publisher, with Elsevier in second place, and together they account for approximately 50% of the journal volume. Among 1,513 co-cited journals, there were 14 journals with more than 100 citations, and only *Clinical Nutrition* had over 500 (citations: 667) (Supplementary Table S2). Interestingly, the top three journals in terms of co-citation were also the only ones in the Q1 JCR division among the top 10 journals in terms of publication volume. In terms of centrality, the *British Journal of Surgery* and *American Journal of Physiology-Endocrinology and Metabolism* ranked first (0.12), followed by *Cochrane Database of Systematic Reviews* (0.11), and *Acta Anaesthesiologica Scandinavica* (0.1). Additionally, it was worth noting that the journals in Supplementary Table S2 were all of high quality and ranked in the Q1/Q2 range in their research categories.

**Table 5:** The top 10 journals in the field of PCL.

**Rank**	**Journal**	**Documents**	**JCR**	**Impact factor**	**Publisher**	**Citations**	**Average citations**
1	Clinical Nutrition	16	Q1	6.6	Churchill Livingstone	907	56.69
2	Journal of Perianesthesia Nursing	15	Q2	1.6	Elsevier Science Inc	115	7.67
3	Bmc Anesthesiology	10	Q2	2.3	Bmc	83	8.30
4	World Journal of Surgery	8	Q2	2.3	Wiley	222	27.75
4	Nutrition	8	Q2	3.2	Elsevier Science Inc	224	28.00
6	Nutrition in Clinical Practice	7	Q3	2.1	Wiley	273	39.00
7	British Journal of Surgery	6	Q1	8.7	Oxford Univ Press	752	125.33
7	Anesthesia and Analgesia	6	Q1	4.6	Lippincott Williams & Wilkins	220	36.67
7	Journal of Parenteral and Enteral Nutrition	6	Q2	3.2	Wiley	130	21.67
7	Asia Pacific Journal of Clinical Nutrition	6	Q4	1.3	HEC Press, Healthy Eating Club Pty Ltd	37	6.17

**Figure 5:**
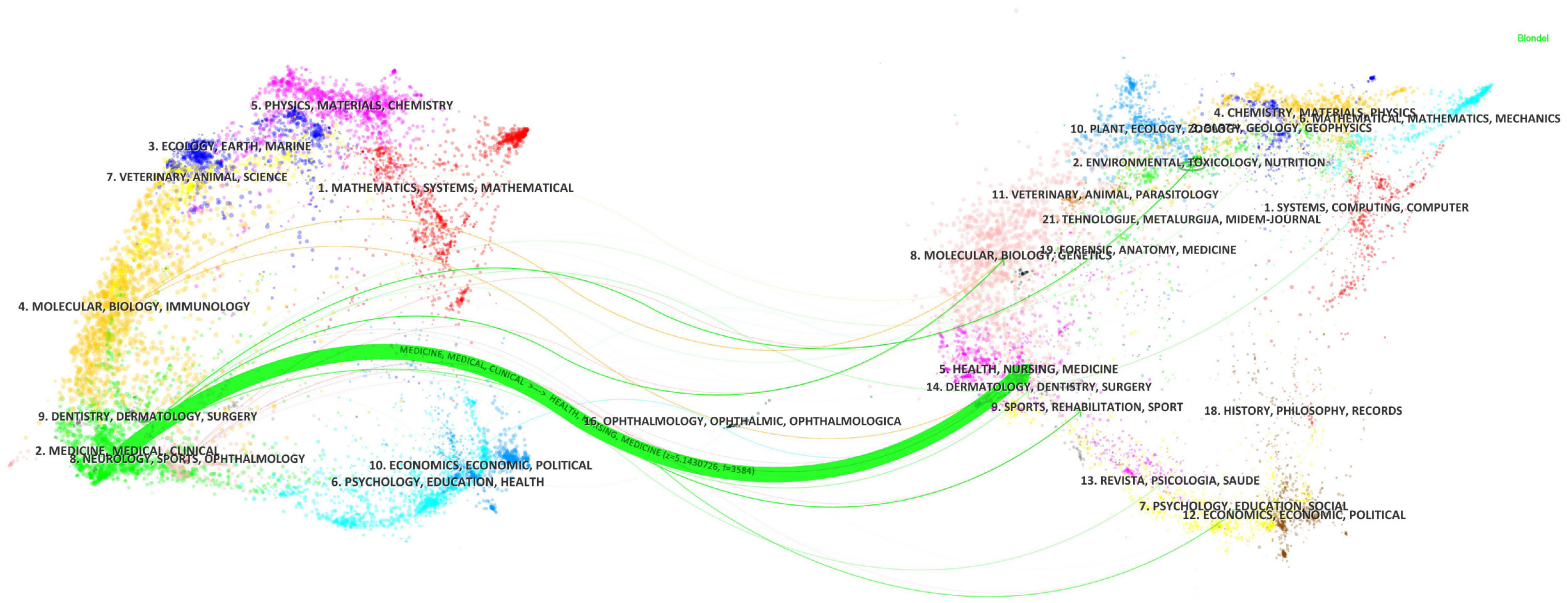
The dual-map overlay.

The following view, shown in Figure 5, illustrates the dual-map overlay visual analytics related to PCL. The distribution of citing journals on the left represented relative domain applications, and the distribution of cited journals on the right referred to the research basis, with the curve representing the citation line in the graph. Only one main citation pathway was present in Figure 5; the green line indicated that articles from Healthy/Nursing/Medicine were mainly cited by articles from Medicine/Medical/Clinical. It can be intuitively predicted that PCL's focal points and frontiers have been focusing on the field of Medicine/Medical/Clinical.

### 3.7 Research hotspot analysis

#### 3.7.1 Keywords profile

**Figure 6:**
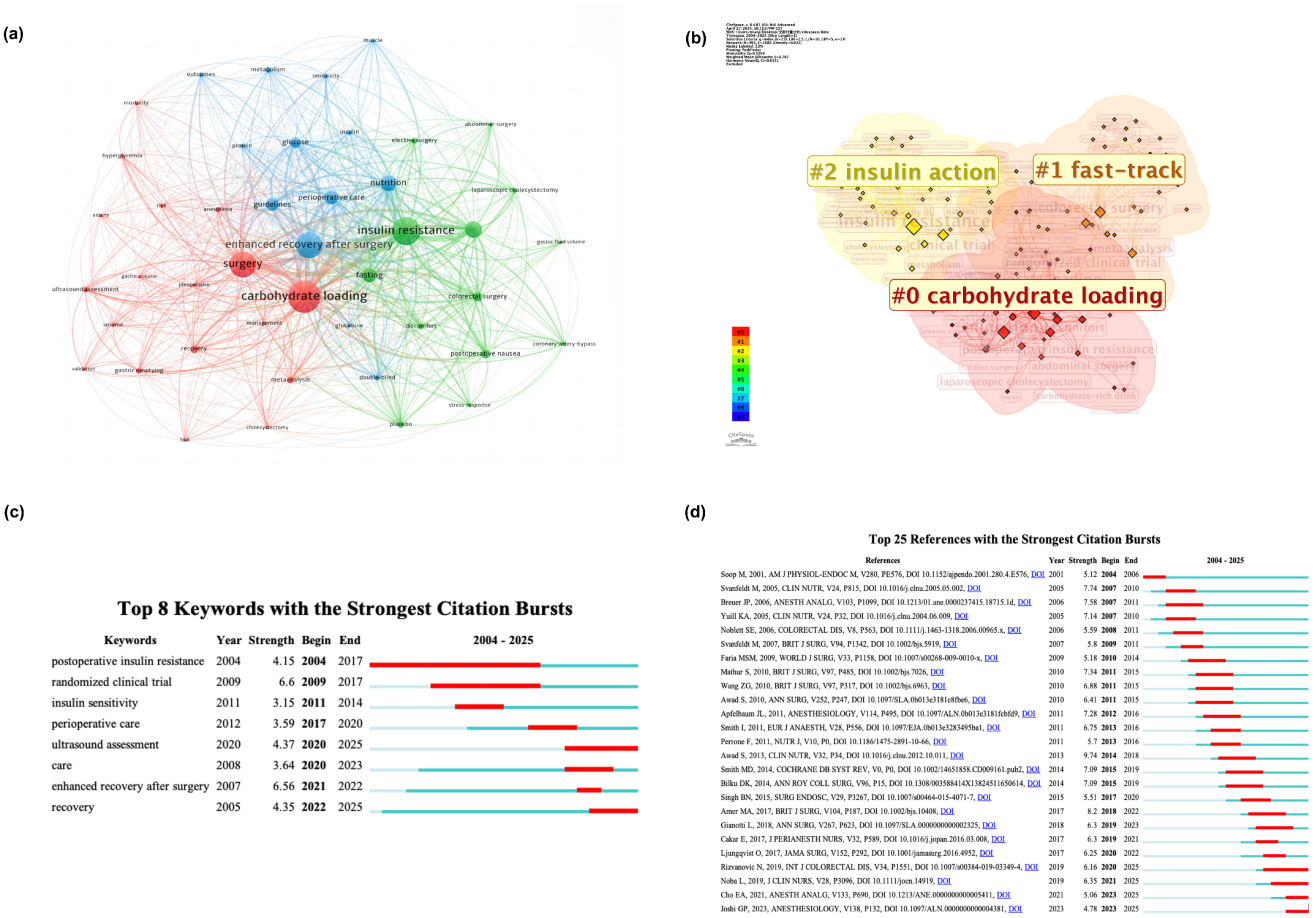
Keyword visualization of cluster mapping and strongest citation bursts. **(a)** The keyword clustering map by VOSviewer; **(b)** The top three keyword clustering map by Citespace; **(c)** Top 8 keywords with the strongest citation bursts. **(d)** Top 25 references with the strongest citation bursts.

Keywords co-occurrence density, clustering and emergence were analyzed to help understand this field's research hotspots, frontiers, and trends. VOSviewer statistics showed 1,014 keywords, of which three appeared 100 times or more, seven appeared 50 times or more, and 15 appeared 20 times or more. The most frequently used keywords were “carbohydrate loading” with 125 occurrences, followed by “insulin resistance” (*n* = 107), “surgery” (*n* = 100), and “ERAS” (*n* = 99). The keywords with the highest centrality were “carbohydrate loading” (*n* = 0.25) and “ERAS” (*n* = 0.25). Those keywords with a frequency of 9 or more times were identified as core keywords according to Lodka's principle, totalling 44 in all. These core keywords were clearly grouped into three clusters (Figure 6a), with the most representative keyword for each group being “carbohydrate loading” (Cluster 1), “insulin resistance” (Cluster 2), and “ERAS” (Cluster 3), respectively. The keywords contained in each group were listed in Table 6. Cluster 2, represented in green, appeared the earliest and focused on the application of PCL in various types of elective surgery, including “abdominal surgery”, “colorectal surgery”, and “coronary-artery-bypass”. Cluster 1, represented in red, focused on the safety of the application of PCL, with keywords such as “gastric emptying”, “gastric volume”, and “ volume”. The newest cluster, Cluster 3, represented in blue, focused on the effects of applying PCL on postoperative recovery, including “enhanced recovery after surgery”, “metabolism”, and “nutrition”. Keyword cluster analysis was also performed using keyword-based logarithmic likelihood ratios using CiteSpace statistics. The modularity Q was 0.5295, and the mean Silhouette score was 0.787. The top three most significant clusters were “carbohydrate loading”, “fast-track”, and “insulin action”, with a beautiful butterfly-shaped cluster map being demonstrated (Figure 6b). There were 12 keywords with a centrality greater than or equal to 0.1, and the top five are “ERAS” (0.27), “carbohydrate loading” (0.26), “guidelines” (0.18), “insulin resistance” (0.16), and “colorectal surgery” (0.15) (Supplementary Table S3).

**Table 6:** The 44 core keywords and three clusters generated by VOSviewer statistics.

**Cluster**	**Color**	**Keywords**
1	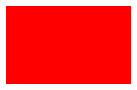	Carbohydrate loading, elderly, preoperative, surgery, cholecystectomy, anesthesia, gastric emptying, gastric volume, volume, hyperglycemia, recovery, mortality, risk, ultrasound assessment, trial, validation, management, meta-analysis
2	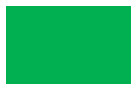	Insulin resistance, randomized clinical trial, elective surgery, abdominal surgery, colorectal surgery, coronary-artery-bypass, laparoscopic cholecystectomy, fasting, placebo, discomfort, gastric fluid volume, postoperative nausea, stress-response
3	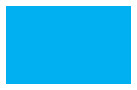	Enhanced recovery after surgery, metabolism, glucose, glutamine, insulin, sensitivity, muscle, protein, nutrition, outcomes, perioperative care, guidelines, double-blind

#### 3.7.2 Keywords bursts

Keyword burst analysis revealed considerable keyword changes in a short period (Figure 6c). The strongest burst keyword was “randomized clinical trial”, with a burst strength reaching 6.6, followed by “ERAS”, with a value of 6.56. The keywords with the strongest bursts revealed that they were hot topics at various stages of PCL development, and we categorized them into three groups based on the time of the burst. The first group of keywords ended with burst before 2017, which was the beginning time of the third phase of literature publication, and included three keywords, “postoperative insulin resistance” (2004–2017), “insulin sensitive” (2011–2014), and “randomized clinical trial” (2009–2017); of them, the keyword “postoperative insulin resistance” not only had the longest lasting burst time (14 years), but also had an burst from the earliest publication year of the literature included in this study. The second and third groups both appeared with a burst after 2017. The difference was that the burst times of Group 3 (Keywords: “ultrasound assessment” and “recovery”) have continued today and might remain as hot keywords for the future.

#### 3.7.3 References, co-cited references, and bursts

**Figure 7:**
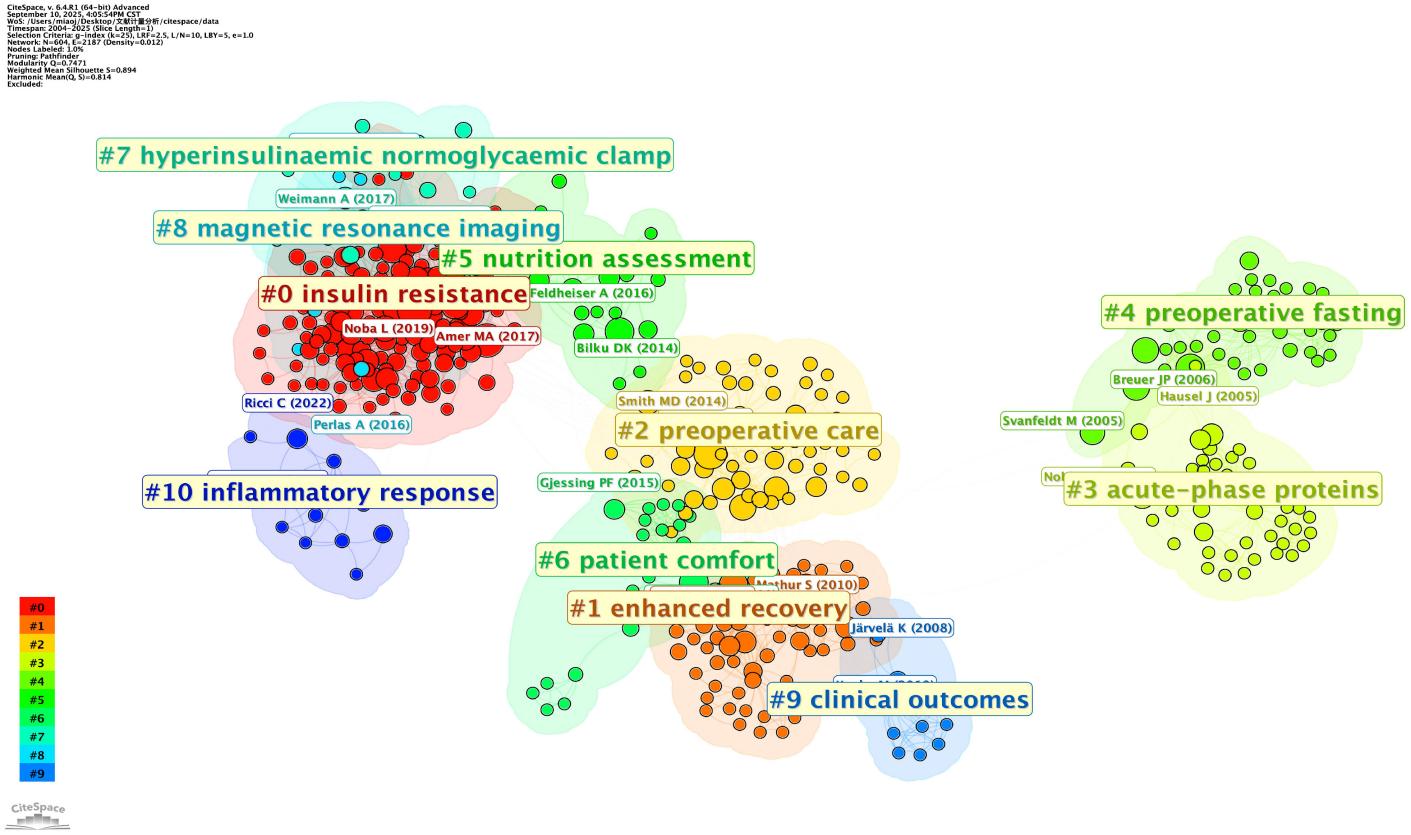
The visualization of the co-citation network.

The top ten most-cited references were published from 1995 to 2014 (Supplementary Table S4). Hausel et al.'s work received the most citations (111), propelling it to the top of the list, and their article was the only one with more than 100 citations. Olle Ljungqvist was the corresponding author of six widely cited references that rank first to fifth, and ninth, respectively. Intriguingly, the top ten cited and co-cited references (2005–2017) barely overlapped (Supplementary Tables S4 and S5), with only Hausel et al.'s research (66 citations, centrality 0.14) and Noblett et al.'s research (60 citations, centrality 0.1) ranked in both lists. Breuer's study had the highest centrality (0.34) among co-cited references, whilst Amer's research had the most co-citations (24). Surprisingly, Pexe-Machado et al.'s research had a centrality of 0.14 with only four co-citations. CiteSpace identified the top 25 references with the strongest citation bursts (Figure 6d). We noticed an intriguing “ceaselessness”: from 2004 to 2025, there had been a constant stream of reference bursts, but the bursts had been short-lived. Four references reported the burst time continuing today, including two randomized controlled trials (RCTs), one systematic review, and one guideline. A total of ten major clusters were recognized (Figure 7).

We divided these clusters into four categories: Metabolic response (#0 insulin resistance, #3 acute-phase proteins, #4 preoperative fasting, and #10 inflammatory response), recovery and clinical outcomes (#1 enhanced recovery, #6 patient comfort, and #9 clinical outcome), Preoperative management (#2 preoperative care, and #5 nutrition assessment), and Reasearch techniques (#7 hyperinsuliaemic normoglucaemic clamp, and #8 magnetic resonanace imaging (MRI)).

#### 3.7.4 Topic and network patterns in PubMed clinical trials

**Figure 8:**
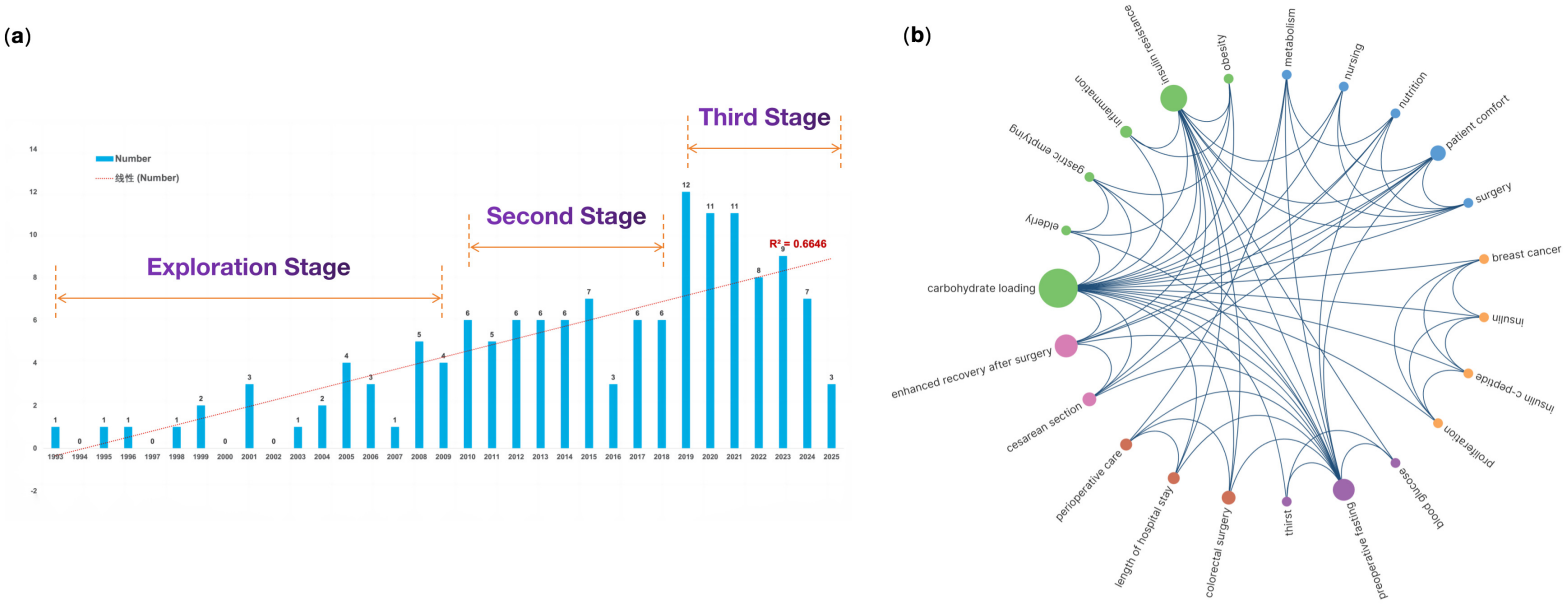
Profile of PubMed-identified clinical trials. **(a)** Trends in the annual publication of clinical trials; **(b)** Keyword co-occurrence.

We conducted a thematic analysis of abstracts from 141 clinical trials sourced from PubMed and constructed keyword co-occurrence (Figure 8). Publications were categorized into three periods based on research theme. In the foundational phase (1993–2000), research demonstrated the fundamental physiological benefits and safety profile of PCL, primarily involving general surgical populations. Outcome measures included safety profiles, basic metabolic parameters, and gastric residual volumes. In the next phase (2001–2010), the scope widened to reveal the metabolic and recovery advantages of PCL across diverse surgical populations, including colorectal and orthopedic surgery. Studies conducted during this period provided some evidence of improved patient insulin sensitivity, reduced postoperative complications such as nausea and vomiting, shorter hospital stays, and enhanced quality of recovery. Since 2011, research has addressed more specialized clinical questions, focusing on vulnerable patient groups such as the elderly and diabetic patients, and those facing major surgery. These investigations have demonstrated how PCL optimizes metabolic pathways, accelerates recovery, and enables personalized, evidence-based preoperative care. The most frequently used keywords were “carbohydrate loading”, “insulin resistance”, and “ERAS”. Meanwhile, the most prominent research clusters focused on the safety and benefits of PCL for specific populations, which align with the results in WoSCC.

#### 3.7.5 Surgical types represented in the PCL literature

The PCL research encompassed a diverse range of surgical types (Supplementary Table S6), reflecting broad clinical interest in perioperative metabolic optimisation, with a clear focus on gastrointestinal, cardiac, and orthopedic domains. Gastrointestinal procedures dominated the literature, with colorectal surgery (33 occurrences) and laparoscopic cholecystectomy (16 occurrences) constituting the most frequently studied operations. This emphasis likely reflects the significant postoperative metabolic consequences of these procedures and their suitability for controlled perioperative nutritional interventions. Cardiac surgical procedures (28 occurrences) appeared with substantial representation, indicating early exploration of PCL in high-risk populations. Orthopedic surgery, particularly hip arthroplasty and total knee arthroplasty, represented the third major procedural category, suggesting recognition of the potential to mitigate stress response in major joint reconstruction. Notably, bariatric and cancer-related surgeries received comparatively limited attention, suggesting emerging rather than established interest in these specialities.

## 4 Discussion

### 4.1 Research Sources

The publication trajectory of PCL demonstrates clear, multi-phase growth. The temporal pattern suggests a transition from hypothesis generation and small trials to broader adoption, protocolisation, and evaluation within ERAS frameworks. During the embryonic stage, clinical trials and concept papers establish physiological principles (attenuating fasting-induced catabolism and mitigating insulin resistance) and safety considerations (30–33). Exploratory small-scale trials and the integration of these concepts into perioperative fasting guidelines characterize the current literature (30). Subsequently, the number and scale of clinical trials increase, and researchers start synthesizing evidence through systematic reviews and guideline-oriented work (34–36). Increased publications from 2017 onward reflect not only more primary studies but also more systematic reviews, meta-analyses, and implementation research addressing pragmatic questions: which patient groups benefit most (1, 3, 37, 38), optimal carbohydrate formulations/timing (9, 39–41), safety across different surgical contexts (e.g., major abdominal, orthopedic, and cardiac surgery) (42–44), and economic and patient-reported outcomes (PROs) (45, 46). The stabilization at higher annual counts after 2020 reflects maturation: PCL has become an established research subfield with sustained interest. Citation trends fluctuate but have generally increased in tandem with publication volume, indicating that the field's outputs are being increasingly referenced within the wider surgical and perioperative literature. The publication and citation profile coincide with the broader adoption of ERAS programs and growing interest in multimodal perioperative optimisation.

The top ten countries account for 82.90% of publications, showing a concentrated global contribution. The People's Republic of China (62 papers) is the largest single contributor, followed by the USA (35), England (23), and Sweden (22). This concentration is typical for many clinical subfields, where research capacity, funding, and clinical trial infrastructure are unevenly distributed. High centrality, but lower raw publication counts (e.g., France, Norway), suggest that these countries punch above their weight through extensive international collaboration. The USA, England, and Sweden combine high output with dense connectivity, fostering knowledge dissemination and multicenter trials (47, 48). China's large node but low connectivity reveals a different pattern: high productivity with limited international collaboration. Factors may include language barriers, focus on local patient populations/contexts, or institutional priorities. Increasing multinational partnerships could enhance methodological rigor, generalizability, and citation impact for China-based research. European countries exhibit a strong influence relative to their size—reflecting longstanding research traditions in perioperative medicine, robust academic networks, and early adoption of ERAS programs. The rising output of Asian countries, combined with weaker collaboration, suggests a next-stage opportunity: integrating into global consortia and multicenter RCTs to increase impact. Overall, though 38 countries contributed, the field remains dominated by high- and upper-middle–income countries. There is limited representation from low- and lower-middle–income countries. This raises issues for external validity: evidence generated in resource-rich settings may not fully translate to low-resource contexts, where perioperative infrastructure, nutritional status, and surgical case mix differ. Future research should aim for more geographically diverse, context-sensitive studies. The institution profile, collaboration network, and centrality metrics reveal a fragmented landscape with limited cross-institutional cohesion. Robust evidence (large RCTs, replication across settings) benefits from multi-institutional collaboration. Fragmented institutional networks may lead to slower accumulation of high-quality multicenter evidence and variable methodological quality and reporting. Moreover, the heterogeneity of evidence and lack of cross-validation complicate the synthesis and formulation of guidelines. Encouraging institutional consortia, shared registries, and international trial networks would accelerate evidence robustness and external validity. The author who makes the most outstanding and irreplaceable contribution is Olle Ljungqvist, who invented the idea of preoperative oral carbohydrates and co-founded the ERAS^®^ Society. He is a pioneer and advocate of the use of PCL instead of overnight fasting for elective surgery (49). The academic team he leads has published the top five highly cited papers and 70% of the top ten highly cited papers (49–55). He and his team member, Jonas Nygren, present the top two productive authors and co-cited authors. Despite the presence of productive teams, overall author centrality in this field is low, implying limited widespread, cross-group collaboration. Notably, 60% of the most productive co-authors and 60% of the top five authors are Swedish, reinforcing Sweden's outsized influence. Such concentration can produce leadership and clarity of thought, but may also bias the literature toward specific clinical paradigms or patient populations. Stronger cross-group collaboration—such as international multicenter RCTs, shared datasets, and coordinated meta-analyses—would likely enhance the field's methodological rigor and external validity. Mentoring networks that connect established authors with emerging investigators, particularly those from underrepresented regions, would diversify perspectives and extend applicability. PCL research is published across 120 journals, but a core of 10 journals accounts for over 30% of the literature. The dual pattern—a modest core set of high-impact journals plus a broad tail of speciality and regional journals—suggests that while seminal research appears in high-visibility venues, a substantial portion of studies are dispersed. This dispersion may hinder rapid synthesis unless systematic reviews actively include gray literature and regional journals. The high centrality of the British Journal of Surgery and the American Journal of Physiology—Endocrinology and Metabolism underscores the interplay between clinical surgery, nutrition science, and metabolic physiology in shaping the field. Based on the overlap between the top ten journals by frequency and centrality, the *British Journal of Surgery and Anesthesia and Analgesia* are the most important and influential journals in this field. Wiley and Elsevier account for 50% of the journal volume, reflecting major publishers‘ role in disseminating PCL research. Our findings are useful for researchers' communication and manuscript submission to appropriate potential journals. Journal selection influences visibility, citations, and clinical uptake. Encouraging publication in high-quality, accessible journals and ensuring indexing in major databases will improve integration into guidelines. However, getting published in these high-quality journals is challenging. Furthermore, we analyzed the types of surgery encountered in PCL studies. These investigations have progressively expanded beyond gastrointestinal, cardiac and orthopedic fields to encompass weight-loss surgery, cancer surgery and other diverse specialities. This heterogeneous procedural distribution indicates that the PCL concept was conceptualized as a broadly applicable perioperative nutrition strategy rather than a procedure-specific intervention during this publication period, further demonstrating the widespread recognition of PCL.

### 4.2 Research Topics

Co-citation analysis and reference bursts provide a better understanding of the research foundation of PCL. These early studies have laid the theoretical and empirical groundwork for subsequent research in this field. We focused on the co-citation clustering results (Figure 7) and combined the most cited and co-cited references (Table S4 and Table S5) to suggest the following research topics:

- **Metabolic responses**. Preoperative fasting (#4) is a traditional practice aimed at reducing the risk of aspiration during anesthesia. However, extended fasting can lead to catabolism and increased insulin resistance (#0) (56, 57). PCL is a valuable strategy to minimize fasting durations while adhering to safety protocols (58). Co-citation clustering (#0), as well as high-cited and high-co-cited references, and keyword clustering all identified “insulin resistance” (#0) as the most dominant research topic in the field of PCL. Not only is “insulin resistance” (#0) the top-ranked co-citation cluster, but it is also the earliest identified and most widely accepted postoperative problem that can be alleviated through PCL (49). Mechanistically, carbohydrate intake stimulates glycogen synthesis in the liver and muscles, providing a readily available energy source while minimizing proteolysis and lipolysis (59). Energy content may be a key determinant of PCL efficacy, potentially influencing which studies report positive vs. neutral outcomes. Studies utilizing lower-energy carbohydrate loads more frequently reported reductions in postoperative insulin resistance and infection rates than higher-dose interventions (1, 2). However, our bibliometric analysis indicates that researchers publishing highly co-cited references tended to use standardized, higher-energy protocols (typically 600 kcal). The variation in energy content may explain some of the contradictory findings in our co-citation analysis, in which certain research clusters yielded divergent conclusions despite similar study designs. Future research should standardize energy delivery to better establish energy-response relationships and optimize clinical protocols.

Furthermore, balancing metabolic benefits and risks (e.g., hyperglycemia in diabetic patients) necessitates careful protocol design. Some studies have noted minimal metabolic improvements in patients with obesity or diabetes, suggesting the need for personalized approaches (38, 60–62). Additionally, the long-term effects of repeated carbohydrate loading in elective surgeries remain understudied. The inflammatory response (#10) to surgery is a double-edged sword: while essential for tissue repair, excessive inflammation prolongs recovery and increases the risk of complications (63). PCL has been shown to temper this response, as evidenced by reduced levels of acute-phase proteins (#3) like C-reactive protein (CRP). Clinical trials report lower postoperative CRP concentrations in patients receiving carbohydrate-rich drinks, suggesting diminished systemic inflammation (4, 41). However, heterogeneity in study designs—such as carbohydrate composition (e.g., maltodextrin vs. glucose) and patient populations—complicates the drawing of universal conclusions (17, 38, 64). Some trials report no significant differences in inflammatory markers, attributing the variability to surgical complexity or comorbidities (64, 65).

- **Recovery and clinical outcomes**. PCL is one of the cornerstones of ERAS pathways (#1), which emphasize multimodal interventions to accelerate recovery (3, 8). As evidenced by the top-cited, top-cocited, and high-centrality references, clinical outcomes (#9) associated with this strategy include reduced postoperative fatigue (17, 66), earlier return of gastrointestinal function (53, 67), shorter hospitalization (32, 68), and improved muscle function (69). These benefits stem from metabolic stabilization (#0), reduced inflammation (#3 & #10), and improved patient tolerance of surgical stress. Clinical outcomes also extend to subjective measures like PROs (#6). Studies have noted higher satisfaction scores in patients receiving preoperative carbohydrates, attributed to reduced thirst, hunger, and discomfort (13, 70). Economically, these improvements result in cost savings through reduced hospital stays. Challenges persist in extrapolating these findings to high-risk populations, such as those with severe malnutrition or metabolic disorders (60). What's more, although our bibliometric analysis indicates that PCL improves postoperative recovery and clinical outcomes, this may be attributable to its role within the ERAS protocol. In fact, ultimate patient outcomes depend largely on the continuity of postoperative nutrition. Unless sufficient amino acids and calories are provided to meet ongoing requirements, lean tissue will be lost rapidly (71, 72). The attenuation of the catabolic state induced by PCL cannot be sustained, let alone influence short- or long-term effects (73).
- **Preoperative management**. Effective preoperative care (#2) is crucial for optimizing patient outcomes, and nutrition assessment is vital in this process. Nutritional assessment (#5) is essential for identifying patients who may benefit most from carbohydrate loading, particularly those at risk of malnutrition (36, 74). Malnourished individuals face heightened risks of complications, making targeted supplementation critical (75). Conversely, overfeeding in well-nourished patients may negate metabolic benefits, underscoring the need for tailored approaches (76). Nutrition assessment also informs the composition and timing of carbohydrate solutions. For instance, patients with diabetes require low-glycemic-index formulations to prevent hyperglycemia, while those with malnutrition benefit from protein-enriched carbohydrates to stimulate muscle protein synthesis (38, 77).
- **Research techniques**. The hyperinsulinaemic normoglucaemic clamp (#7) is a research technique used to evaluate insulin sensitivity and metabolic responses to PCL. This method enables researchers to precisely measure the impact of carbohydrate intake on insulin dynamics and overall metabolic health (78). MRI (#8) can further aid in assessing the physiological changes carbohydrate loading induces. By leveraging these diagnostic tools, healthcare providers can gain a comprehensive understanding of how PCL affects individual patients, ultimately guiding the development of tailored interventions that promote optimal surgical outcomes.

### 4.3 Frontiers and future directions

Research frontiers are generally inferred by analyzing the evolution of keyword bursts and end years. However, we found that relying solely on high-frequency keyword data is insufficient, potentially overlooking some high-quality research. As a result, we combined burst keywords and keyword clusters with a co-cited reference timeline map (Figure 6) (79). The frontiers can be categorized as follows.

- **Personalisation of PCL protocols**. The high-frequency keywords such as “elderly,” “coronary-artery-bypass”, and “cholecystectomy” indicate a diverse patient population undergoing various surgical procedures. Current protocols typically adopt a generalized approach to PCL, which may overlook individual patient conditions and responses. The literature reflects variability in outcomes based on patient demographics, comorbidities, and surgical types, suggesting that a one-size-fits-all approach may not be the most effective (2, 8, 38). Future research may be dedicated to: 1) tailored protocols: research should focus on developing personalized PCL protocols based on individual patient characteristics, including age, metabolic status, comorbidities (e.g., diabetes, obesity), and the type of surgery being performed. For instance, elderly patients or those with metabolic disorders may require modified carbohydrate formulations or adjusted dosing to optimize their metabolic responses and minimize risks (38, 77). 2) diverse formulations: investigate the effects of different carbohydrate formulations (e.g., glucose, maltodextrin, or mixed carbohydrates with protein) on metabolic outcomes and recovery. The impact of these formulations on insulin sensitivity, gastric emptying, and overall postoperative recovery can be explored through RCTs specifically designed to assess these variables across different patient profiles. 3) patient engagement: incorporating patient preferences and experiences into the PCL protocol can enhance adherence and outcomes (80). Future studies could evaluate the impact of patient education on the benefits of carbohydrate loading, potentially leading to improved compliance and satisfaction.
- **Mechanistic insights into metabolic responses and inflammatory responses**. The burst keywords “insulin sensitivity,” “postoperative insulin resistance,” and high-frequency keywords “hyperglycemia” and “protein” and burst preference highlight the need for a deeper understanding of the underlying mechanisms through which carbohydrate loading influences metabolic pathways and inflammatory responses. While many studies have demonstrated favorable outcomes, the precise biological mechanisms remain inadequately explored. 1) biomarker research: conduct studies utilizing biomarkers to elucidate the biochemical pathways affected by carbohydrate loading. This includes assessing how preoperative carbohydrate intake influences inflammatory cascades and metabolic stress responses during the perioperative period. 2) longitudinal studies: implement longitudinal studies to evaluate how carbohydrate loading impacts metabolic responses over time. Monitoring these changes at various time points can provide insights into the duration and persistence of metabolic benefits. 3) gut microbiome influence: investigate the potential role of the gut microbiome in mediating the effects of carbohydrate loading on postoperative outcomes. The relationship between dietary carbohydrates, gut health, and immune function could reveal novel insights into enhancing recovery.
- **Harmonization of outcome measures**. The variability in reported outcomes across studies, including hospital stay, insulin resistance, and postoperative discomfort, suggests a need for harmonization to assess the impact of carbohydrate loading. Harmonization can facilitate comparisons across studies and improve the generalizability of findings. The potential research topics include: 1) core outcome sets: develop and validate a core outcome set for trials investigating preoperative carbohydrate loading (81). This should consist of key metrics such as metabolic markers, recovery times, PROs, and safety endpoints. 2) harmonization of protocols: encourage the establishment of standardized protocols for carbohydrate loading in clinical practice. This would facilitate comparison across studies and improve the implementation of best practices in diverse clinical settings. 3) multicenter trials: promote multicenter trials that adopt standardized protocols to assess the effects of carbohydrate loading across different institutions and populations. This can enhance the robustness of the findings and support the development of evidence-based guidelines.
- **Integration of technology in monitoring and implementation**. The references to ultrasound assessment for gastric volume and the evolving monitoring tools suggest potential for technological integration in preoperative care. Utilizing technology can improve patient safety and optimize carbohydrate loading protocols. Researchers may be interested in: (1) real-time monitoring: investigate the feasibility of using non-invasive technologies, such as ultrasound, to monitor gastric volume and emptying in real-time. This can help refine the timing of carbohydrate administration and ensure patient safety. In addition, ambulatory blood glucose monitoring systems (ABGMS), including continuous glucose monitors and flash glucose monitoring devices, could play a crucial role in real-time assessment of glycemic responses to PCL, particularly in patients with diabetes or insulin resistance. These technologies enable clinicians to monitor glucose fluctuations throughout the perioperative period, potentially allowing for individualized carbohydrate dosing and timing adjustments, and the observation of postoperative outcomes. (2) digital health solutions: explore the role of digital platforms and mobile applications for patient education and adherence to preoperative carbohydrate loading protocols. Enhancing patient engagement through technology can improve compliance and outcomes.

The future research frontiers in PCL are poised to enhance the understanding and application of this intervention in surgical care. By focusing on the personalisation of protocols, mechanistic insights, harmonization of outcomes, and technological integration, researchers can address current knowledge gaps and optimize patient outcomes across diverse surgical populations. Continued exploration in these areas aligns with ongoing trends in perioperative care and supports the broader goal of improving recovery experiences and clinical effectiveness in surgical practices.

### 4.4 Limitation

This bibliometric study had some limitations. Despite developing thorough and prudent search strategies and attempting to include as much of the literature published in various formats as possible, we were only able to retrieve articles from the WoSCC database and clinical trials from the PubMed database. The articles not included in WoSCC and clinical trials not included in PubMed may have been overlooked. However, full reference texts and citation lists are not available in most databases, such as Embase, MEDLINE, and Google Scholar. Therefore, we were unable to cover all publications on this topic. As we all know, bibliometric research is primarily based on co-citations. Because of the potential for bias, our findings should be interpreted with caution. Due to the aforementioned potential biases, including inadequate citations, delayed citations, and recent research trends, among others, it may be challenging to detect specific publications (79).

## 5 Conclusion

This study provides, for the first time, a broad overview of existing research on PCL, along with valuable insights into future research directions. The bibliometric patterns indicate that, despite substantial progress in understanding metabolic responses and perioperative management, key gaps remain in translating PCL evidence into personalized, harmonized, and technologically supported clinical nutrition practices. Although emerging work has begun to explore individual variability in metabolic and inflammatory responses, most clinical protocols still rely on fixed-dose, non–individualized regimens that overlook differences in metabolic health, surgical stress exposure, and nutritional risk. Likewise, the absence of harmonized outcome measures continues to limit comparability across studies and hinders the development of unified clinical guidelines. Research techniques are evolving toward continuous monitoring and mechanistic characterization, yet these tools are not routinely applied in real–world perioperative pathways. Given these gaps, clinicians and dietitians should prioritize tailoring PCL strategies to patient-specific factors, while integrating simple metabolic monitoring tools, such as ABGMS, to better assess individual responses. Harmonized documents of outcome measures should be routinely adopted to align practice with emerging research. Finally, multidisciplinary collaboration between surgeons, anesthetists, and dietitians is essential to ensure consistent implementation, with digital platforms and mobile applications to improve adherence and facilitate ongoing audit of protocol effectiveness.

## Data Availability

The raw data supporting the conclusions of this article will be made available by the authors, without undue reservation.
