# Supplementary material for: Preoperative carbohydrate loading: evolution, trends, and future directions
Source: Front Nutr. 2026 Mar 11;13:1750029. doi: 10.3389/fnut.2026.1750029 (PMC13012943; doi:10.3389/fnut.2026.1750029)
Supplement: Supplementary file 1 [file Table_1.docx]

**Search strategy for PubMed**

#1 "preoperative period"[MeSH Terms] 10265

#2 "preoperative care"[MeSH Terms] 74878

#3 "pre?surgical"[Title/Abstract] 4427

#4 "perioperative period"[MeSH Terms] 108273

#5 "perioperative care"[MeSH Terms] 163652

#6 "perioperative nursing"[MeSH Terms] 14132

#7 "perioperative medicine"[MeSH Terms] 183

#8 "surg*"[Title/Abstract] 2622222

#9 "operat*"[MeSH Terms] 117129

#10 #1 OR #2 OR #3 OR #4 OR #5 OR #6 OR #7 OR #8 OR #9 2820371

#11 carbohydrates[MeSH Terms] 2536034

#12 "CHO"[Title/Abstract] 44161

#13 "maltodextrin"[Title/Abstract] 3644

#14 #11 OR #12 OR #13 2573287

#15 "load*"[Title/Abstract] 575977

#16 "drink*"[Title/Abstract] 182207

#17 "beverage*"[MeSH Terms] 184794

#18 "treatment*"[Title/Abstract] 6102921

#19 "administration*"[Title/Abstract] 1048441

#20 "oral"[Title/Abstract] 783390

#21 "per os"[Title/Abstract] 6306

#22 #15 OR #16 OR #17 OR #18 OR #19 OR #20 OR #21 7854262

#23 #10 AND #14 AND #22 38206

# 24 limited article type to [clinical trial] 5971
