# Supplementary material for: Preoperative carbohydrate loading: evolution, trends, and future directions
Source: Front Nutr. 2026 Mar 11;13:1750029. doi: 10.3389/fnut.2026.1750029 (PMC13012943; doi:10.3389/fnut.2026.1750029)
Supplement: Supplementary file 2 [file Table_2.docx]

Supplementary Material 2

# Table S1: The top 10 co-authors in the field of PCL

| **RANK** | **AUTHOR** | **CITATIONS** | **CENTRALITY** | **MAIN ORGANISATION** | **COUNTRY** |
| --- | --- | --- | --- | --- | --- |
| 1 | Jonas Nygren | 308 | 0.14 | Karolinska Institutet | Sweden |
| 2 | Olle Ljungqvist | 245 | 0.08 | Örebro University/Karolinska Institutet | Sweden |
| 3 | J Hausel | 178 | 0.16 | Ersta Hospital/Karolinska Institutet | Sweden |
| 4 | Anders Thorell | 146 | 0.07 | Karolinska Institutet | Sweden |
| 5 | Mattias Soop | 121 | 0.03 | Karolinska Institute | Sweden |
| 6 | Sherif S Awad | 119 | 0.09 | Minia University | Egypt |
| 7 | U O Gustafsson | 95 | 0.07 | Ersta Hospital | Sweden |
| 8 | Monika Svanfeldt | 93 | 0.03 | Karolinska Institutet | Sweden |
| 9 | A Perlas | 69 | 0.05 | Toronto Western Hospital | Canada |
| 10 | Mads Gram Henriksen | 68 | 0.08 | Aarhus University Hospital | Aarhus |

**Table S2:** The top 14 co-journals in the field of PCL

| **RANK** | **SOURCE** | **CITATIONS** | **CENTRALITY** | **IF** | **JCR** | **PUBLISHER** |
| --- | --- | --- | --- | --- | --- | --- |
| 1 | Clinical Nutrition | 667 | 0.06 | 6.6 | Q1 | Churchill Livingstone |
| 2 | British Journal of Surgery | 471 | 0.12 | 8.7 | Q1 | Oxford Univ Press |
| 3 | Anaesthesia and Analgesia | 428 | 0.08 | 4.6 | Q1 | Lippincott Williams & Wilkins |
| 4 | British Journal of Anaesthesia | 273 | 0.03 | 9.1 | Q1 | Elsevier Sci Ltd |
| 5 | Anesthesiology | 269 | 0.09 | 9.3 | Q1 | Lippincott Williams & Wilkins |
| 6 | Annals of Surgery | 264 | 0.07 | 7.9 | Q1 | Lippincott Williams & Wilkins |
| 7 | Acta Anaesthesiologica Scandinavica | 250 | 0.1 | 1.9 | Q2 | Wiley |
| 8 | World Journal of Surgery | 161 | 0.06 | 2.3 | Q2 | Wiley |
| 9 | Nutrition | 150 | 0.07 | 3.2 | Q2 | Elsevier Science Inc |
| 10 | Cochrane Database of Systematic Reviews | 143 | 0.11 | 8.8 | Q1 | Wiley |
| 11 | American Journal of Physiology-Endocrinology and Metabolism | 132 | 0.12 | 4.2 | Q1 | Amer Physiological Soc |
| 12 | Journal Of Parenteral and Enteral Nutrition | 131 | 0.09 | 3.2 | Q2 | Wiley |
| 13 | European Journal of Anaesthesiology | 130 | 0.06 | 4.2 | Q1 | Lippincott Williams & Wilkins |
| 14 | Best Practice & Research-Clinical Anaesthesiology | 118 | 0.02 | 4.7 | Q1 | Elsevier |

**Table S3:** The top 44 core keywords in the field of PCL

| **RANK** | **KEYWORDS** | **OCCURRENCES** | **CENTRALITY** |
| --- | --- | --- | --- |
| 1 | Carbohydrate Loading | 125 | 0.25 |
| 2 | Insulin Resistance | 107 | 0.19 |
| 3 | Surgery | 100 | 0.06 |
| 4 | Enhanced Recovery After Surgery | 99 | 0.25 |
| 5 | Randomised Clinical Trial | 60 | 0.01 |
| 6 | Nutrition | 57 | 0.14 |
| 7 | Fasting | 51 | 0 |
| 8 | Perioperative Care | 47 | 0.01 |
| 9 | Guidelines | 44 | 0.19 |
| 10 | Glucose | 36 | 0.2 |
| 11 | Colorectal Surgery | 33 | 0.16 |
| 12 | Postoperative Nausea | 30 | 0.01 |
| 13 | Recoveny | 27 | 0.04 |
| 14 | Meta analysis | 21 | 0.06 |
| 14 | Placebo | 21 | 0.03 |
| 16 | Gastric Emptying | 19 | 0.06 |
| 16 | Elective Surgery | 19 | 0.04 |
| 18 | Double-Blind | 18 | 0.08 |
| 18 | Discomfort | 18 | 0.04 |
| 18 | Ultrasound Assessment | 18 | 0.02 |
| 18 | Metabolism | 18 | 0.02 |
| 22 | Outcomes | 16 | 0.02 |
| 22 | Laparoscopic Cholecystectomy | 16 | 0.01 |
| 22 | Insulin | 16 | 0.01 |
| 25 | Anesthesia | 15 | 0.06 |
| 26 | Sensitivity | 14 | 0.01 |
| 27 | Abdominal-surgery | 13 | 0.04 |
| 27 | Protein | 13 | 0.01 |
| 27 | Stress-response | 13 | 0.01 |
| 27 | Hypergiycemia | 13 | 0.01 |
| 27 | Muscle | 13 | 0 |
| 32 | Coronary-artery-bypass | 12 | 0.02 |
| 32 | Volume | 12 | 0.01 |
| 32 | Risk | 12 | 0.01 |
| 32 | Management | 12 | 0.01 |
| 36 | Mortality | 11 | 0.01 |
| 37 | Glutamine | 10 | 0 |
| 38 | Preoperative | 9 | n/a |
| 38 | Elderly | 9 | n/a |
| 38 | Gastric volume | 9 | 0.02 |
| 38 | Gastric fluid volume | 9 | 0.02 |
| 38 | Cholecystectomy | 9 | 0 |
| 38 | Validation | 9 | 0 |
| 38 | Trial | 9 | 0 |

**Table S4:** The top 10 cited references in the field of PCL.

| **RANK** | **REFERENCE** | **CITATIONS** | **CENTRALITY** |
| --- | --- | --- | --- |
| 1 | Hausel J, Nygren J, Lagerkranser M, et al. A carbohydrate-rich drink reduces preoperative discomfort in elective surgery patients. Anesth Analg. 2001;93(5):1344-1350. doi:10.1097/00000539-200111000-00063 | 111 | 0 |
| 2 | Nygren J, Thorell A, Jacobsson H, et al. Preoperative gastric emptying. Effects of anxiety and oral carbohydrate administration. Ann Surg. 1995;222(6):728-734. doi:10.1097/00000658-199512000-00006 | 79 | n/a |
| 3 | Nygren J, Soop M, Thorell A, Efendic S, Nair KS, Ljungqvist O. Preoperative oral carbohydrate administration reduces postoperative insulin resistance. Clin Nutr. 1998;17(2):65-71. doi:10.1016/s0261-5614(98)80307-5 | 71 | 0 |
| 3 | Soop M, Nygren J, Myrenfors P, Thorell A, Ljungqvist O. Preoperative oral carbohydrate treatment attenuates immediate postoperative insulin resistance. Am J Physiol Endocrinol Metab. 2001;280(4):E576-E583. doi:10.1152/ajpendo.2001.280.4.E576 | 71 | 0 |
| 5 | Hausel J, Nygren J, Thorell A, Lagerkranser M, Ljungqvist O. Randomised clinical trial of the effects of oral preoperative carbohydrates on postoperative nausea and vomiting after laparoscopic cholecystectomy. Br J Surg. 2005;92(4):415-421. doi:10.1002/bjs.4901 | 66 | 0.14 |
| 6 | Henriksen MG, Hessov I, Dela F, Hansen HV, Haraldsted V, Rodt SA. Effects of preoperative oral carbohydrates and peptides on postoperative endocrine response, mobilisation, nutrition and muscle function in abdominal surgery. Acta Anaesthesiol Scand. 2003;47(2):191-199. doi:10.1034/j.1399-6576.2003.00047.x | 64 | 0.01 |
| 7 | Yuill KA, Richardson RA, Davidson HI, Garden OJ, Parks RW. The administration of an oral carbohydrate-containing fluid prior to major elective upper-gastrointestinal surgery preserves skeletal muscle mass postoperatively--a randomised clinical trial. Clin Nutr. 2005;24(1):32-37. doi:10.1016/j.clnu.2004.06.009 | 62 | 0.14 |
| 8 | Noblett SE, Watson DS, Huong H, Davison B, Hainsworth PJ, Horgan AF. Preoperative oral carbohydrate loading in colorectal surgery: a randomised controlled trial. Colorectal Dis. 2006;8(7):563-569. doi:10.1111/j.1463-1318.2006.00965.x | 60 | 0.1 |
| 9 | Thorell A, Nygren J, Ljungqvist O. Insulin resistance: a marker of surgical stress. Curr Opin Clin Nutr Metab Care. 1999;2(1):69-78. doi:10.1097/00075197-199901000-00012 | 59 | 0 |
| 9 | Smith MD, McCall J, Plank L, Herbison GP, Soop M, Nygren J. Preoperative carbohydrate treatment for enhancing recovery after elective surgery. Cochrane Database Syst Rev. 2014;2014(8):CD009161. Published 2014 Aug 14. doi:10.1002/14651858.CD009161.pub2 | 59 | 0.05 |

**Table S5:** The top 10 co-cited references in the field of PCL.

| **RANK** | **REFERENCE** | **CO-CITATIONS** | **CENTRALITY** |
| --- | --- | --- | --- |
| 1 | Amer MA, Smith MD, Herbison GP, Plank LD, McCall JL. Network meta-analysis of the effect of preoperative carbohydrate loading on recovery after elective surgery. Br J Surg. 2017;104(3):187-197. doi:10.1002/bjs.10408 | 24 | 0.26 |
| 2 | Noba L, Wakefield A. Are carbohydrate drinks more effective than preoperative fasting: A systematic review of randomised controlled trials. J Clin Nurs. 2019;28(17-18):3096-3116. doi:10.1111/jocn.14919 | 22 | 0.05 |
| 2 | Rizvanović N, Nesek Adam V, Čaušević S, Dervišević S, Delibegović S. A randomised controlled study of preoperative oral carbohydrate loading versus fasting in patients undergoing colorectal surgery. Int J Colorectal Dis. 2019;34(9):1551-1561. doi:10.1007/s00384-019-03349-4 | 22 | 0.01 |
| 4 | Awad S, Varadhan KK, Ljungqvist O, Lobo DN. A meta-analysis of randomised controlled trials on preoperative oral carbohydrate treatment in elective surgery. Clin Nutr. 2013;32(1):34-44. doi:10.1016/j.clnu.2012.10.011 | 20 | 0.14 |
| 4 | Gianotti L, Biffi R, Sandini M, et al. Preoperative Oral Carbohydrate Load Versus Placebo in Major Elective Abdominal Surgery (PROCY): A Randomised, Placebo-controlled, Multicenter, Phase III Trial. Ann Surg. 2018;267(4):623-630. doi:10.1097/SLA.0000000000002325 | 20 | 0.03 |
| 6 | Mathur S, Plank LD, McCall JL, et al. Randomised controlled trial of preoperative oral carbohydrate treatment in major abdominal surgery. Br J Surg. 2010;97(4):485-494. doi:10.1002/bjs.7026 | 16 | 0.16 |
| 7 | Liu B, Wang Y, Liu S, et al. A randomised controlled study of preoperative oral carbohydrate loading versus fasting in patients undergoing elective craniotomy. Clin Nutr. 2019;38(5):2106-2112. doi:10.1016/j.clnu.2018.11.008 | 15 | 0.06 |
| 7 | American Society of Anesthesiologists Committee. Practice guidelines for preoperative fasting and the use of pharmacologic agents to reduce the risk of pulmonary aspiration: application to healthy patients undergoing elective procedures: an updated report by the American Society of Anesthesiologists Committee on Standards and Practice Parameters. Anesthesiology. 2011;114(3):495-511. doi:10.1097/ALN.0b013e3181fcbfd9 | 15 | 0.06 |
| 7 | Wang ZG, Wang Q, Wang WJ, Qin HL. Randomised clinical trial to compare the effects of preoperative oral carbohydrate versus placebo on insulin resistance after colorectal surgery. Br J Surg. 2010;97(3):317-327. doi:10.1002/bjs.6963 | 15 | 0.06 |
| 7 | Çakar E, Yilmaz E, Çakar E, Baydur H. The Effect of Preoperative Oral Carbohydrate Solution Intake on Patient Comfort: A Randomised Controlled Study. J Perianesth Nurs. 2017;32(6):589-599. doi:10.1016/j.jopan.2016.03.008 | 15 | 0.04 |

**Table S6:** Surgical types represented in the PCL literature.

| **Keywords** | | | **Frequency** | | |
| --- | --- | --- | --- | --- | --- |
| **Abdominal Surgery:** | | | **15** |  |  |
| abdominal surgery | |  | 14 |  | 15 |
|  | major abdominal-surgery | | 1 |  |  |
| **Cardiac Surgery:** | | | **28** |  |  |
| cardiac surgery | | | 8 |  |  |
|  | myocardial revascularization | | 17 | 20 |  |
|  |  | coronary artery bypass graft | 3 |  |  |
| **Gastrointestinal Surgery:** | | | **77** |  |  |
| gastrointestinal surgery | | | 7 |  | 77 |
|  | colorectal surgery | | 33 |  |  |
|  | cholecystectomy | | 9 | 25 |  |
|  |  | laparoscopic cholecystectomy | 16 |  |  |
|  | colonic surgery | | 6 |  |  |
|  | gastrectomy | | 2 |  |  |
|  | hepatectomy | | 1 |  |  |
|  | pancreaticoduodenectomy | | 1 |  |  |
|  | pancreatic surgery | | 1 |  |  |
|  | small-bowel resection | | 1 |  |  |
| **Laparoscopic Procedures:** | | | **8** |  |  |
| laparoscopic surgery |  |  | 4 |  | 8 |
|  | laparoscopic colon cancer surgery |  | 1 |  |  |
|  | laparoscopic nephrectomy |  | 1 |  |  |
|  | day care laparoscopic cholecystectomy |  | 1 |  |  |
|  | gynecological laparoscopic surgery |  | 1 |  |  |
| **Orthopedic Surgery:** | | | **26** |  |  |
| orthopedic surgery |  |  | 2 |  | 26 |
|  | arthroplasty |  | 5 | 22 |  |
|  |  | hip arthroplasty | 12 |  |  |
|  |  | total knee arthroplasty | 4 |  |  |
|  |  | total joint arthroplasty | 1 |  |  |
|  | lumbar disc surgery |  | 1 |  |  |
|  | spinal surgery |  | 1 |  |  |
| **Urological Surgery:** | | | **6** |  |  |
| nephrectomy |  |  | 1 |  |  |
| cystectomy |  |  | 1 |  |  |
| radical cystectomy |  |  | 1 |  |  |
| radical prostatectomy |  |  | 3 |  |  |
| **Bariatric Surgery:** |  |  | **8** |  |  |
| bariatric surgery |  |  | 3 |  |  |
| gastric bypass |  |  | 1 | 3 |  |
|  | roux-en-y gastric bypass |  | 2 |  |  |
| sleeve gastrectomy |  |  | 2 |  |  |
| **Cancer Surgery:** | | | **14** |  |  |
| oncologic surgery |  |  | 1 |  |  |
| debulking surgery |  |  | 1 |  |  |
| breast cancer |  |  | 2 |  |  |
| colorectal cancer |  |  | 1 | 2 |  |
|  | colon cancer |  | 1 |  |  |
| gastric cancer |  |  | 1 |  |  |
| liver-cancer |  |  | 1 | 3 |  |
|  | hepatocellular carcinoma |  | 2 |  |  |
| head and neck cancer |  |  | 1 |  |  |
| oral cancer |  |  |  |  |  |
| gynecologic cancer |  |  | 1 | 2 |  |
|  | surgical gynaecological cancer | | 1 |  |  |
| epithelial ovarian cancer |  |  | 1 |  |  |
| **Obstetric and Gynecologic Surgery:** | | | **12** |  |  |
| caesarean delivery |  |  | 10 |  |  |
| benign gynecologic surgery | | | 1 |  |  |
| myomectomy |  |  | 1 |  |  |
| **Other Specific Procedures:** | | | **7** |  |  |
| thyroidectomy |  |  | 1 |  |  |
| elective craniotomy |  |  | 1 |  |  |
| transplantation |  |  | 1 | 2 |  |
|  | kidney transplant |  | 1 |  |  |
| vascular-surgery |  |  | 1 |  |  |
| lower extremity bypass |  |  | 1 |  |  |
| thoracic surgery |  |  | 1 |  |  |
